# Retrograde Suppression of Post-Tetanic Potentiation at the Mossy Fiber-CA3 Pyramidal Cell Synapse

**DOI:** 10.1523/ENEURO.0450-20.2021

**Published:** 2021-03-10

**Authors:** Sachin Makani, Stefano Lutzu, Pablo J. Lituma, David L. Hunt, Pablo E. Castillo

**Affiliations:** 1 Dominick P. Purpura Department of Neuroscience, Albert Einstein College of Medicine, Bronx, NY 10461; 2 Department of Psychiatry and Behavioral Sciences, Albert Einstein College of Medicine, Bronx, NY 10461

**Keywords:** calcium, hippocampus, mossy fiber, presynaptic, retrograde signaling, short-term plasticity

## Abstract

In the hippocampus, the excitatory synapse between dentate granule cell (GC) axons, or mossy fibers (MFs), and CA3 pyramidal cells (MF-CA3) expresses robust forms of short-term plasticity, such as frequency facilitation and post-tetanic potentiation (PTP). These forms of plasticity are due to increases in presynaptic neurotransmitter release, and can be engaged when dentate GCs fire in bursts (e.g., during exploratory behaviors) and bring CA3 pyramidal neurons above threshold. While frequency facilitation at this synapse is limited by endogenous activation of presynaptic metabotropic glutamate receptors (mGluRs), whether MF-PTP can be regulated in an activity-dependent manner is unknown. Here, using physiologically relevant patterns of MF stimulation in acute mouse hippocampal slices, we found that disrupting postsynaptic Ca^2+^ dynamics increases MF-PTP, strongly suggesting a form of Ca^2+^-dependent retrograde suppression of this form of plasticity. PTP suppression requires a few seconds of MF bursting activity and Ca^2+^ release from internal stores. Our findings raise the possibility that the powerful MF-CA3 synapse can negatively regulate its own strength not only during PTP-inducing activity typical of normal exploratory behaviors, but also during epileptic activity.

## Significance Statement

The powerful mossy fiber (MF)-CA3 synapse exhibits strong forms of plasticity that are engaged during location-specific exploration, when dentate granule cells (GCs) fire in bursts. While this synapse is well-known for its presynaptically expressed long-term potentiation (LTP) and long-term depression (LTD), much less is known about the robust changes that occur on a shorter time scale. How such short-term plasticity is regulated, in particular, remains poorly understood. Unexpectedly, a GC in vivo like pattern of activity induced robust presynaptically-expressed post-tetanic potentiation (PTP) only when the postsynaptic cell was loaded with a high concentration of Ca^2+^ buffer, indicating a form of Ca^2+^-dependent retrograde suppression of PTP. Such suppression may have profound implications for how environmental cues are encoded into neural assemblies, and for limiting network hyperexcitability during seizures.

## Introduction

Mossy fibers (MFs), the axonal projections of dentate granule cells (GCs), provide a strong excitatory input onto hippocampal CA3 pyramidal neurons (Henze et al., 2000; Nicoll and Schmitz, 2005). The MF-CA3 synapse is well known for exhibiting uniquely strong forms of short-term potentiation, including paired-pulse facilitation (PPF) and frequency facilitation, which last milliseconds to seconds (Salin et al., 1996). More intense periods of high-frequency stimulation typically elicit post-tetanic potentiation (PTP; e.g., MF-PTP), which decays over several minutes (Griffith, 1990). These forms of plasticity are commonly attributed to an increase in presynaptic release probability (Pr; Zucker and Regehr, 2002; Nicoll and Schmitz, 2005; Regehr, 2012), a change in the readily releasable pool (Vandael et al., 2020), and transiently convert the synapse from a high-pass to lower-pass filter (Abbott and Regehr, 2004). In behaving rodents, during place field activation, GCs can fire in high-frequency bursts (Pernía-Andrade and Jonas, 2014; Diamantaki et al., 2016; GoodSmith et al., 2017; Senzai and Buzsáki, 2017), driving CA3 pyramidal neurons above threshold (Henze et al., 2002). Thus, frequency facilitation and MF-PTP could have a profound impact on how memory traces are encoded into CA3 neural ensembles.

Given the apparent ease with which these robust forms of presynaptic potentiation are elicited at the MF-CA3 synapse, one might expect a process by which the connection is negatively regulated. In fact, there is evidence that endogenously released glutamate transiently suppresses frequency facilitation via presynaptic Group II metabotropic glutamate receptors (II-mGluRs; Scanziani et al., 1997; Toth et al., 2000; Kwon and Castillo, 2008a). It is unknown, however, whether MF-PTP is also curtailed in an activity-dependent manner.

In the present study we performed whole-cell recordings from CA3 pyramidal neurons in mouse hippocampal slices and mimicked physiologically relevant activity patterns by stimulating GCs with a brief, high-frequency bursting paradigm. To our surprise, MF-PTP was readily observed when the postsynaptic cell was dialyzed with a solution containing high Ca^2+^ buffering properties, but was nearly absent when the recording solution had a lower, more physiological Ca^2+^ buffering capacity. These findings strongly suggest that MF-PTP is normally suppressed by a Ca^2+^-dependent retrograde mechanism. Such negative feedback could not only enable homeostatic regulation of MF-CA3 synaptic strength, ensuring that the essential filtering properties of the connection are maintained during ongoing activity, but also prevent runaway network excitability, as may occur during epileptic activity.

## Materials and Methods

### Slice preparation

Animal procedures were approved by our Institutional Animal Care and Use Committee and adhered to National Institutes of Health guidelines. C57BL6 mice of either sex (18–28 d old) were deeply anesthetized with isoflurane, decapitated, and brains rapidly removed and both hippocampi were dissected. Transverse hippocampal slices (400 μm) were cut on a DTK-2000 microslicer (Dosaka) or a Leica VT1200 S vibratome, in ice-cold cutting solution containing the following: 215 mm sucrose, 2.5 mm KCl, 20 mm glucose, 26 mm NaHCO_3_, 1.6 mm NaH_2_PO_4_, 1 mm CaCl_2_, 4 mm MgSO_4_, and 4 mm MgCl_2_. After 10 min of incubation at room temperature, the cutting solution was exchanged for the artificial CSF (ACSF) containing 124 NaCl, 2.5 KCl, 10 glucose, 26 NaHCO_3_, 1 NaH_2_PO_4_, 2.5 CaCl_2_, and 1.3 MgSO_4_. Both cutting and ACSF solutions were saturated with 95% O_2_ and 5% CO_2_ (pH 7.4). The slices recovered at room temperature for at least 1.5 h before recording.

### Electrophysiology

**Figure 1.**
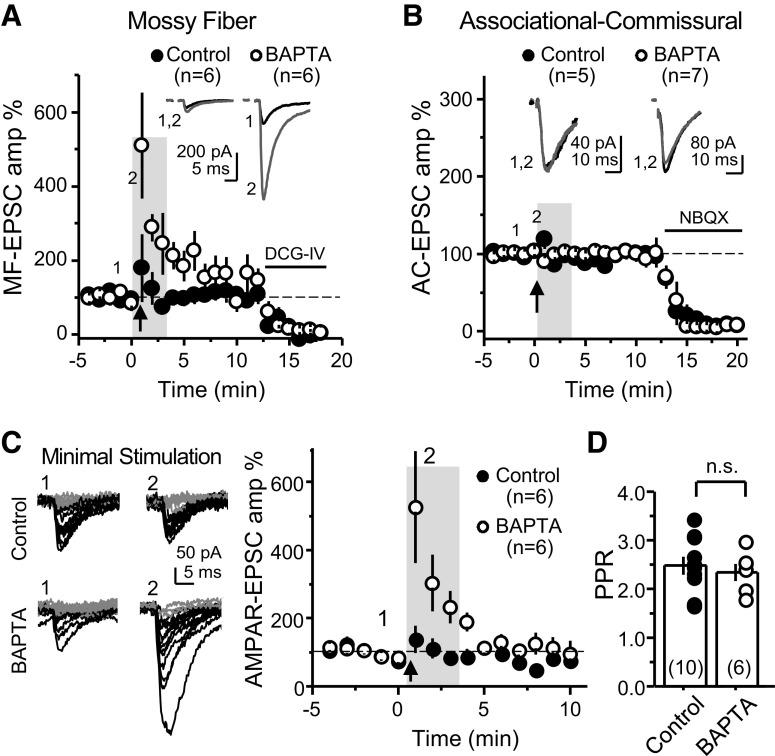
High postsynaptic Ca^2+^ buffering selectively enhances PTP at the MF-CA3 synapse. ***A***, Summary data showing the effect of loading the patch pipette with 10 mm BAPTA on PTP of AMPAR-mediated MF-responses compared with control condition (0.1 mm EGTA; control: 125 ± 52% of baseline; *n* = 6; BAPTA: 347 ± 84%; *n* = 6; control vs BAPTA: *p* < 0.05). Experiments were performed at 32°C, Vh = −60 mV, with only a low concentration of the AMPAR selective antagonist GYKI-53655 (1 μm), and the bursting paradigm was performed in current-clamp mode. AMPAR-EPSCs were evoked with bulk stimulation in the GC layer. At the end of the experiments, DCG-IV (1 μm) was added to the bath to verify that the synaptic responses were mediated by MF activation. PTP is quantified as the average of the EPSCs during the first 3 min postinduction versus the 3-min prior induction. Representative traces (top) and time course summary plot (bottom). ***B***, Summary data of experiments in which the same bursting paradigm was performed at the neighboring associational-commissural (CA3-CA3) synapse. Note no difference between control and BAPTA-dialyzed cells (control: 101 ± 6%; *n* = 5; BAPTA: 95 ± 3%; *n* = 7; control vs BAPTA: *p* > 0.3). NBQX was used at the end of the experiments to confirm AMPAR-mediated responses. ***C***, PTP assessed by minimal stimulation of MFs while monitoring AMPAR-EPSCs in the absence of drugs in the bath. PTP was induced in current-clamp mode. Left, Representative traces showing successes (black) and failures (gray). Right, Summary data showing MF-PTP elicited with minimal stimulation in control and BAPTA-dialyzed cells (control: 112 ± 27 of baseline; *n* = 6; BAPTA: 354 ± 82; *n* = 6; control vs BAPTA: *p* < 0.05). Gray box indicates the 3-min post-PTP time window quantified for comparison in control and BAPTA conditions in all experiments. ***D***, PPR was not affected by intracellular 10 mm BAPTA loading (control: 2.5 ± 0.18; *n* = 10; BAPTA: 2.3 ± 0.17; *n* = 6; control vs BAPTA: *p* > 0.5). Number of cells are indicated between brackets. Here and in all figures, data are presented as mean ± SEM; representative traces correspond to the time points indicated by numbers on the time course plots. n.s., non-significant.

Slices were transferred to a recording chamber and perfused with ACSF (2 ml/min). Recordings were done at 25°C, except for the experiments included in Figures 1*A*, 6*E–H*, and those that used botulinum toxin-B (BoTX) that were performed at 32°C. The recording pipette was filled with an internal solution containing the following: 112 mm K-gluconate, 17 mm KCl, 0.04 mm CaCl_2_, 10 mm HEPES, 2 mm Mg-ATP, 0.2 mm GTP, 10 mm NaCl, and 0.1 mm EGTA (pH 7.2; 290–295 mOsm). For the experiments using 10 mm EGTA or BAPTA, the concentration of K-gluconate was reduced to compensate for osmolarity. The pipette resistance ranged from 3 to 4 MΩ. Series resistance (6–15 MΩ) was monitored throughout the experiment, and those experiments in which the series resistance changed by >10% were not included for analysis. Patch pipettes were pulled on a PP-830 vertical puller (Narishige).

Responses were generated by stimulating presynaptic axons and recording from CA3 pyramidal neurons. Kainate receptor (KAR) responses were recorded in the presence of GYKI-53655 (30 μm) or LY 303070 (15 μm), and CGP-55845 (3 μm) in the bath, and MK-801 (2 mm) in the pipette. With the exception of data obtained in Figures 1*A*, 6*E–H*; Extended Data Figure 5-1*C*, AMPA receptor (AMPAR)-mediated responses were also generated with MK-801 in the pipette, and GYKI-53655 (1 μm) in the bath (Kwon and Castillo, 2008a). NMDA receptor (NMDAR) responses were recorded with NBQX (10 μm) in the bath. All experiments except those in Figures 1*A*,*C*, 6*E–H* contained picrotoxin (100 μm).

10.1523/ENEURO.0450-20.2021.f5-1 Extended Data Figure 5-1 ***A***, Bath application of the Group I mGluR agonist DHPG (50 μm) caused an inward current, was significantly reduced in the presence of the Group I mGluR antagonists MPEP/CPCCOEt (control: 55 ± 11 pA; *n* = 5; MPEP/CPCCOEt: 14 ± 5 pA; *n* = 4; control vs antagonists: *p* < 0.05). Inset, Superimposed traces in control and in the presence of MPEP/CPCCOEt. Calibration bars: 40 pA, 1 min. ***B***, The outward current induced by bath application of the GABA_B_ agonist baclofen was abolished by intracellular loading of GDP-βS (control: 61 ± 19 pA; *n* = 8; GDP-βS: 3 ± 6 pA; *n* = 6; control vs GDP-βS: *p* < 0.05). Inset, Superimposed representative traces in control cells (black) and cells loaded with GDP-βS. Calibration bars: 40 pA, 1 min. ***C***, Loading BoTX in CA1 pyramidal neurons blocked LTP of AMPAR-mediated transmission (control: 240 ± 18% of baseline; *n* = 5; BoTX: 137 ± 30%; *n* = 5; control vs BoTX: *p* < 0.05). LTP was generated using a pairing protocol of postsynaptic depolarization (from V_H_ = –60–0 mV) for 3 min, and stimulating the Schaffer collaterals with 180 pulses (2 Hz). ***D***, Positive control showing the immediate potentiating effect of 200 nm DPCPX (221 ± 25% of baseline, *n* = 4; p < 0.01). ***E***, The suppression of MF-mediated responses by 1 μm DCG-IV bath application was reversed by the antagonists LY341495 (1 μm) and MSOP (200 μm; 90 ± 24% of original baseline; *n* = 4). Download Figure 5-1, EPS file.

KAR and NMDAR-mediated EPSCs were evoked by placing a monopolar stimulating pipette with a broken tip (∼5–10 μm in diameter, filled with ACSF) in the dentate gyrus (DG) cell body layer. For AMPAR-mediated responses, the tip was left unbroken (∼1 μm) to minimize the number of MFs activated. For minimal stimulation experiments, a theta-glass stimulating pipette was placed in stratum lucidum 100 μm away from the recorded CA3 pyramidal cell. Intensity was increased until a success/failure pattern of AMPAR-EPSC responses was observed. MF AMPAR-EPSCs were only accepted for analysis if the following criteria were met: robust PPF (at least 2-fold), DCG-IV sensitivity >85%, fast rise time (10–90%) was <1.2 ms, response onset was <5.0 ms, and these values did not significantly change after the bursting. These criteria were based on those established by previous studies of MF-CA3 transmission (Jonas et al., 1993). To record KAR-EPSCs and AMPAR-EPSCs, cells were voltage-clamped at −60 mV. For NMDAR-EPSCs, cells were held at −50 mV. Unless otherwise stated, baseline NMDAR-EPSCs and KAR-EPSCs were obtained by delivering two stimuli separated by 5 ms to evoke a measurable response (Weisskopf and Nicoll, 1995). Stimulus intensity for KAR/NMDAR-mediated and AMPAR-mediated responses was ∼100 and 10 μA, respectively. Stimulus intensities did not differ, on average, between control and BAPTA-dialyzed cells in any given condition. Stimulus duration was 100–200 μs. CA3 neurons were always dialyzed for ≥ 15 min, while stimulating at 0.1 Hz, before delivering any plasticity-inducing stimulation. For synaptically evoked action potentials (Fig. 6*E–H*), no drugs were bath applied and CA3 pyramidal cells were held in current-clamp mode before, during and after the induction. Resting potential was kept between −60 and −75 mV. Baseline and postinduction spiking probability were measured as the average of number of spikes per burst normalized to the number of pulses per burst. (i.e., three pulses at 25 Hz).

**Figure 2.**
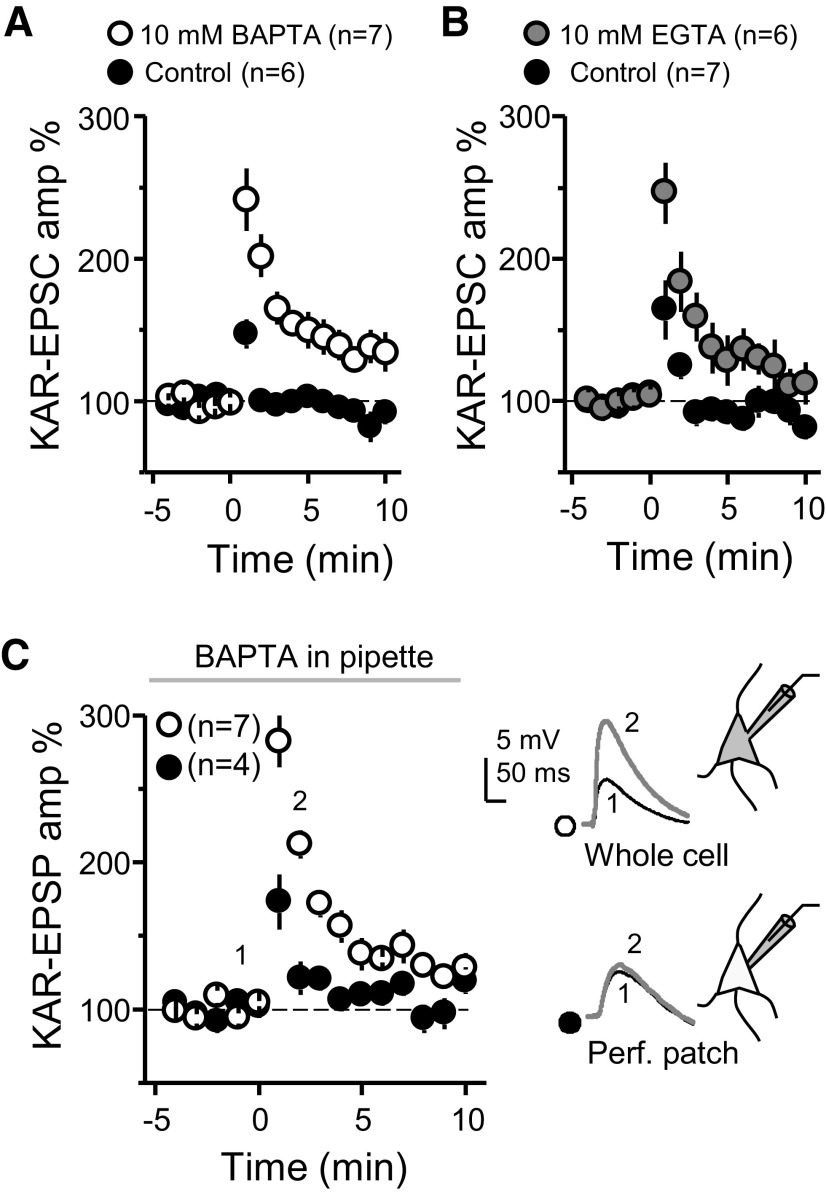
Weak MF-PTP of KAR-mediated transmission under physiological postsynaptic Ca^2+^ buffering recording conditions. ***A***, Summary data showing the effect of 10 mm BAPTA on MF-PTP (control: 115 ± 6%; *n* = 6; BAPTA: 203 ± 14%; *n* = 7; control vs BAPTA: *p* < 0.001). ***B***, Summary data for control versus 10 mm EGTA (control: 122 ± 13%, *n* = 7; 10 mm EGTA: 196 ± 17% of baseline, *n* = 6; control vs 10 mm EGTA *p* < 0.01). ***C***, left, Summary effect when 10 mm BAPTA was included in the recording pipette, in whole-cell versus perforated-patch configuration (BAPTA perforated patch: 138 ± 11%; *n* = 7; BAPTA whole-cell: 226 ± 11%; *n* = 4; BAPTA perforated patch vs BAPTA whole-cell: *p* < 0.001). Right, EPSPs from representative experiments. See Extended Data Figure 2-1 for supporting information.

**Figure 3.**
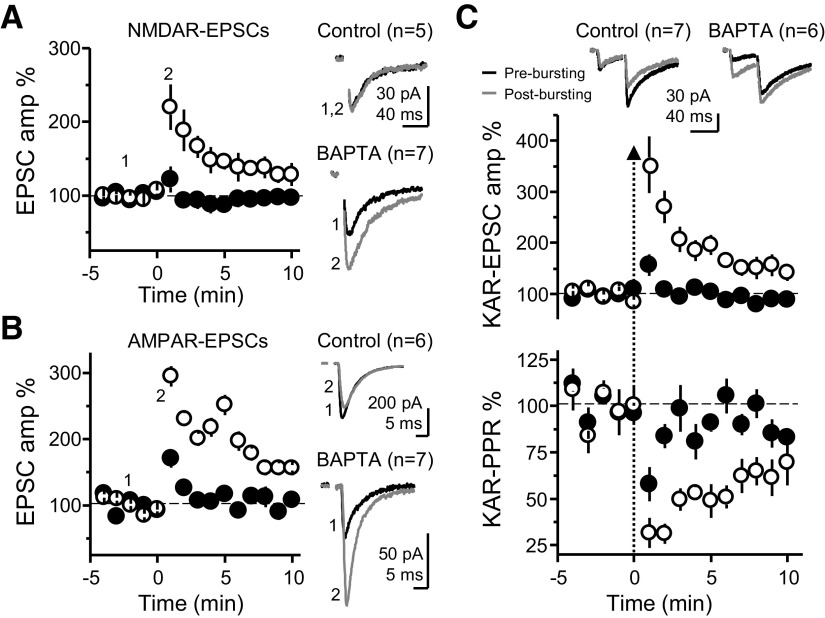
Enhanced MF-PTP by high postsynaptic Ca^2+^ buffering capacity is presynaptically expressed. ***A***, Summary data showing the effect of 25 bursts on NMDAR-mediated EPSCs (V_H_ = −50 mV), when CA3 neurons were dialyzed with control versus BAPTA intracellular solution (control: 102 ± 11% of baseline; *n* = 5; BAPTA: 191 ± 23%; *n* = 7; control vs BAPTA: *p* < 0.05). ***B***, Summary plot of the AMPAR-mediated MF response after the bursting paradigm, in control versus BAPTA conditions (control: 135 ± 17%; *n* = 6; BAPTA: 223 ± 16%; *n* = 7; control vs BAPTA: *p* < 0.01). ***C***, KAR PPR was monitored by delivering two pulses (40-ms interstimulus interval). Top, Averaged traces from representative experiments, before and after bursting, and summary plot of the first EPSC amplitude in control versus BAPTA solutions. Bottom, Summary of PPR time course as normalized to the 3 min before the bursting paradigm. Same cells as in top panel (control: 80 ± 8%; *n* = 7; BAPTA: 37 ± 5%; *n* = 6; control vs BAPTA: *p* < 0.01).

The baseline stimulation frequency for all experiments was 0.1 Hz, except for frequency facilitation (five stimuli, 25 Hz), which was delivered at 0.05 Hz. The standard PTP induction protocol consisted of 25 bursts (five stimuli, 50 Hz) delivered at 2 Hz. PTP was never generated more than once in a given slice. To achieve a similar time course of potentiation in BAPTA-dialyzed cells when the interstimulus interval was 40 ms (Fig. 3*C*), or when monitoring MF AMPAR-mediated transmission (Figs. 1*A*, 3*B*), it was necessary to use 50 bursts. The bursting induction protocol was delivered while cells were voltage-clamped at −60 mV, except for the experiments included in Figures 1*A*,*C*, 2*C*, 6*E–H*, in which PTP was induced in current-clamp. Long-term potentiation (LTP) in CA1 pyramidal neurons was induced by pairing postsynaptic depolarization from −60 to 0 mV for 3 min with low-frequency stimulation of Schaffer collaterals (180 pulses, 2 Hz).

In all experiments examining MF synaptic transmission, the mGluR2/3 agonist DCG-IV (1–2 μm) was applied at the end of the experiment and data were included only when the response was inhibited by >85%. For perforated patch experiments, nystatin was first dissolved into DMSO (10 mg/ml). This was then diluted 250-fold into the intracellular solution to yield 40 μg/ml. BoTX was prepared by making a 0.5 μm stock solution with 1 mg/ml BSA. This was then diluted 100-fold into the final intracellular solution, with 0.5 mm DL-dithiothreitol (DTT).

### Reagents

MK-801, NBQX, CGP-55845, nimodipine, DCG-IV, and GYKI 53655 were obtained from Tocris-Cookson. LY 303070 was obtained from ABX advanced biochemical compounds. BoTX was obtained from List Biological. All other chemicals and drugs were purchased from Sigma-Aldrich.

### Data analysis

Experiments were executed with a MultiClamp 700B amplifier (Molecular Devices). Data were analyzed online using IgorPro (Wavemetrics), and offline with Origin 9.2 and GraphPad Prism. The 3 min before the induction protocol was used as a baseline for statistics. Following the protocol, the first 3 min were used to calculate PTP in all experiments of the study, and the last 3 min (of a 30 min period) for the positive control experiment testing LTP at the Schaffer collateral to CA1 pyramidal cell synapse (Extended Data Fig. 5-1*C*). Representative responses are averages of 18 traces. All values are shown as mean ± SEM. Unless otherwise stated, Student’s *t* test was used for statistical significance between two samples, and ANOVA for multiple comparisons. Data that did not display a normal distribution using the Shapiro-Wilk test were compared using the non-parametric test Mann–Whitney and Wilcoxon signed-ranked test for unpaired and paired conditions respectively. All experiments for a given condition were performed in an interleaved fashion, i.e., control experiments were performed for every test experiment.

## Results

### MF PTP is minimal under physiological postsynaptic Ca^2+^ buffering conditions

This study was initiated by the unexpected observation that MF-PTP magnitude was highly dependent on the postsynaptic Ca^2+^ buffering conditions. We induced MF-PTP by activating MFs with a bursting protocol (25 bursts delivered at 2 Hz; five stimuli at 50 Hz within a burst) designed to mimic physiological activity patterns of GCs *in vivo* (Henze et al., 2002; Pernía-Andrade and Jonas, 2014; Diamantaki et al., 2016; GoodSmith et al., 2017; Senzai and Buzsáki, 2017), while monitoring AMPAR-EPSCs (see Materials and Methods) under physiological recording conditions, e.g., no drugs in the bath, near-physiological recording temperature (32°C) and voltage clamping at resting membrane potential (chloride reversal potential). The PTP induction protocol was delivered in current-clamp configuration so that CA3 cells were able to fire freely. To our surprise, we did not observe much potentiation when postsynaptic CA3 pyramidal cells were loaded with 0.1 mm EGTA, a near physiological intracellular Ca^2+^ buffering condition that we refer to as “control,” but saw robust MF-PTP with 10 mm BAPTA in the postsynaptic pipette (Fig. 1*A*). In contrast, BAPTA did not increase PTP of associational-commissural (AC) synaptic responses (Fig. 1*B*). These results are consistent with previous studies that reported little potentiation at the AC synapse (Salin et al., 1996), and suggest that the robust PTP unmasked in BAPTA-dialyzed cells is specific to the MF-CA3 synapse. Importantly, the enhancement of MF-PTP under high postsynaptic Ca^2+^ buffering conditions was also observed with minimal stimulation of MFs (Jonas et al., 1993; Fig. 1*C*), indicating that the PTP enhancement is not an artifact of strong extracellular stimulation. Lastly, we found that 10 mm BAPTA did not affect the basal paired-pulse ratio (PPR; i.e., before bursting; Fig. 1*D*), making unlikely that changes in basal Pr could account for the PTP enhancement. These initial observations suggested that MF-PTP, a phenomenon widely believed to be presynaptic in nature, was under the control of a postsynaptic Ca^2+^-dependent process that deserved further investigation.

A major problem when studying MF-CA3 synaptic plasticity is the polysynaptic contamination associated with extracellular stimulation of MFs (Claiborne et al., 1993; Henze et al., 2000; Nicoll and Schmitz, 2005; Kwon and Castillo, 2008a). Repetitive stimulation aggravates this problem as MF-CA3 synapses can be potentiated several-fold by strong frequency facilitation (Regehr et al., 1994; Salin et al., 1996). To rule out the possibility that the PTP was the result of polysynaptic contamination, we blocked AMPA and NMDAR-mediated transmission, and monitored KAR-EPSCs, which are observed at MF-CA3 synapses but not AC-CA3 synapses (Castillo et al., 1997). When CA3 pyramidal neurons were loaded with 10 mm BAPTA the burst stimulation protocol caused a robust PTP of KAR-mediated transmission as compared with control (0.1 mm EGTA; Fig. 2*A*). To discard that PTP suppression could result from some unexpected effect of BAPTA, we repeated our experiments with 10 mm EGTA, a slow Ca^2+^ chelator that is widely used at this concentration in voltage-clamp recordings. MF-PTP was equally robust in 10 mm EGTA-loaded cells (Fig. 2*B*; Extended Data Fig. 2-1*A*,*B*). To ensure fast postsynaptic Ca^2+^ chelation, subsequent experiments compared cells loaded with 0.1 mm EGTA versus 10 mm BAPTA. Lastly, we verified that KAR-EPSC and AMPAR-EPSC amplitudes in control and BAPTA-loaded cells were comparable (Extended Data Fig. 2-1*C*) and that MF-PTP in BAPTA cells returned to baseline 20 min postinduction (Extended Data Fig. 2-1*D*). Collectively, these findings demonstrate that postsynaptic Ca^2+^ buffering had a striking influence on the magnitude of MF-PTP.

10.1523/ENEURO.0450-20.2021.f2-1 Extended Data Figure 2-1 ***A***, Representative experiment in which the recording pipette solution contained 10 mm EGTA. KAR-EPSC traces, which correspond to the numbers in the time course plot below, are shown above. DCG-IV (1 μm) was added at the end of the experiment. ***B***, Representative experiment in which the pipette solution contained 0.1 mm EGTA (control). Note suppression of PTP relative to ***A***. ***C***, Raw EPSC values of KAR and AMPAR-mediated MF transmission in the presence of EGTA or BAPTA (control KAR EPSC: –45.87 ± 4.7 pA; *n* = 26; BAPTA KAR EPSC: –44.72 ± 4.4 pA; *n* = 28; *p* > 0.8; control AMPAR EPSC = –47.46 ± 10.5 pA; *n* = 10; BAPTA AMPAR EPSC: –49.58 ± 11.51 pA; *n* = 10; *p* > 0.8). ***D***, PTP induction in BAPTA-loaded CA3 pyramidal cells returns to baseline (17- to 20-min KAR EPSC: 102.14 ± 6.1% vs baseline; *n* = 8; *p* > 0.7; paired *t* test). Download Figure 2-1, EPS file.

One interpretation of the set of observations above is that CA3 pyramidal neurons normally have high Ca^2+^ buffering capacity (i.e., similar to 10 mm EGTA), and replacing these with the 0.1 mm EGTA solution somehow abolished PTP. To directly address this possibility, we monitored KAR-EPSPs with BAPTA in the recording pipette, in perforated patch versus whole-cell configuration. In perforated patch conditions, in which BAPTA could not diffuse into the cell, only a small amount of PTP was seen, while KAR-EPSPs in whole-cell mode displayed similar striking potentiation observed above when KAR-EPSCs were recorded from BAPTA-loaded cells (Fig. 2*C*, see also Fig. 2*A*). These results indicate that the endogenous Ca^2+^ buffering capacity of CA3 pyramidal neurons was functionally more similar to the 0.1 mm EGTA solution than to the 10 mm BAPTA solution.

We next confirmed the MF-PTP observed in BAPTA conditions was presynaptic in nature. If this PTP was presynaptic, it should be similarly observed when monitoring KAR-EPSCs, NMDAR-EPSCs and AMPAR-EPSCs (unlike the data reported in Fig. 1*A*, AMPAR-EPSCs in these experiments were pharmacologically isolated and recorded under conditions of low network excitability; see Materials and Methods). Indeed, when either NMDAR-mediated or AMPAR-mediated EPSCs were monitored, little to no potentiation was observed in control conditions, but the response was markedly increased in BAPTA (Fig. 3*A*,*B*). Notably, there was no difference when comparing the PTP magnitude of control cells in KAR, AMPAR, and NMDAR groups to each other (ANOVA; *F* = 1.76; *p* > 0.2, df = 15), or when comparing the BAPTA-dialyzed cells in the three receptor groups to each other (ANOVA; *F* = 0.74; *p* > 0.4, df = 17). Thus, the difference in PTP between control and BAPTA persisted regardless of which postsynaptic receptor was pharmacologically isolated. To assess changes in PPR, we monitored KAR-mediated responses. Here, the induction was accompanied by a greater reduction in PPR in BAPTA-dialyzed cells, consistent with an increase in Pr. Importantly, in both control and BAPTA-dialyzed cells, the recovery time course of EPSC amplitude and PPR mirrored each other (Fig. 3*C*), suggestive of a causal relationship between presynaptic Pr and the postsynaptic response magnitude. Together, these independent lines of evidence confirm a presynaptic locus of MF-PTP in BAPTA-dialyzed cells. Again, as for AMPAR-EPSCs (Fig. 1*D*), no significant difference was noted in the degree of basal PPR (i.e., before bursting) between control and BAPTA-dialyzed cells (control: 2.4 ± 0.2; *n* = 25; BAPTA 2.7 ± 0.2; *n* = 27; control vs BAPTA *p* > 0.2; data not shown), supporting the notion that postsynaptic BAPTA loading did not affect basal Pr. The most parsimonious explanation for a robust presynaptic potentiation being unmasked by preventing a rise in postsynaptic Ca^2+^ is a retrograde signal that suppresses potentiation in normal conditions. This interpretation is consistent with the fact that most forms of retrograde signaling so far described require a rise in postsynaptic Ca^2+^ concentration (Fitzsimonds and Poo, 1998; Regehr et al., 2009).

### Source of postsynaptic Ca^2+^ rise involved in PTP suppression

**Figure 4.**
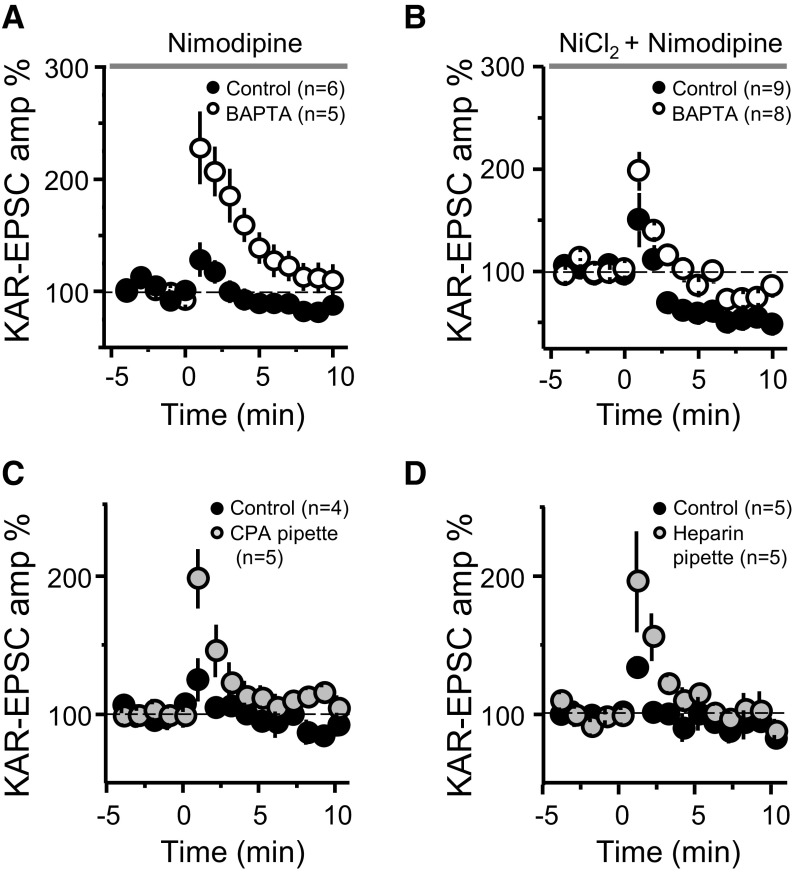
Suppression of MF-PTP depends on internal Ca^2+^ stores. ***A***, Summary effect of 10 μm nimodipine in the bath in control and BAPTA-dialyzed cells (control: 115.24 ± 9.9%; *n* = 6; BAPTA: 201.31 ± 27.8%; *n* = 5; control vs BAPTA: *p* < 0.05). ***B***, Summary effect of adding 100 μm Ni^2+^ and 10 μm nimodipine to the bath (control: 110 ± 14; *n* = 9; BAPTA: 151 ± 9; *n* = 8; control vs BAPTA: *p* < 0.05). ***C***, Summary effect of including CPA in the patch pipette (control: 112 ± 6%; *n* = 4; CPA: 156 ± 16%; *n* = 5; control vs CPA: *p* < 0.05). ***D***, Summary effect of including heparin in the recording pipette to block IP_3_Rs (control: 112 ± 2%; *n* = 5; heparin: 157 ± 17%; *n* = 5; control vs heparin: *p* < 0.05).

We next sought to assess the source or sources of the postsynaptic Ca^2+^ rise involved in the suppression of MF-PTP. Ca^2+^ influx via NMDARs, or Ca^2+^-permeable AMPARs or KARs, was ruled out as solely sufficient for retrograde suppression, given that pharmacological blockade of these receptors did not unmask PTP (Figs. 2, 3*A*). Several voltage-gated Ca^2+^ channels (VGCCs) exist in the thorny excrescence of CA3 pyramidal neurons (Kapur et al., 2001; Reid et al., 2001). Since the thorny excrescences in our experiments could have been poorly voltage-clamped (at −60 mV) during the induction protocol, one or more of these channels may have contributed to the rise in postsynaptic Ca^2+^. To address this possibility, we added nimodipine (10 μm) to the bath solution to ensure L-type VGCCs were blocked in this experiment. If L-type channels were in fact the source of the Ca^2+^ required for the putative retrograde suppression, we would expect that blocking those channels would mimic the effect of BAPTA, and that both control and BAPTA-dialyzed cells exhibit robust PTP. However, under these recording conditions, a large difference between control and BAPTA-dialyzed cells remained (Fig. 4*A*), suggesting that L-type channels do not contribute significantly to the regulation of MF-PTP. We next examined the potential role of R-type and T-type VGCCs by adding 100 μm Ni^2+^. Under these recording conditions, and likely because of the blockade of presynaptic R-type channels, as previously reported (Breustedt et al., 2003; Dietrich et al., 2003), MF-PTP was dampened, but the difference between low and high postsynaptic Ca^2+^ buffering conditions remained (Fig. 4*B*). Intriguingly, MF-PTP in the presence of Ni^2+^ and nimodipine was followed by synaptic depression, but intracellular BAPTA was still able to enhance PTP. It is therefore unlikely that R-type and T-type VGCCs in CA3 pyramidal neurons contribute significantly to MF-PTP suppression.

To investigate the potential role of intracellular Ca^2+^ stores, we included cyclopiazonic acid (CPA; 30 μm) in the patch pipette to deplete Ca^2+^ from the endoplasmic reticulum. CPA led to an increase in MF-PTP relative to control cells (Fig. 4*C*), suggesting that the rise in Ca^2+^ required by the retrograde signal is mediated, at least partially, by intracellular stores. CPA could have diffused from the recorded cell to the presynaptic terminal, reduced Pr, and thereby increased the magnitude of PTP. To address this possibility, we delivered a synaptic burst (five pulses, 25 Hz) to cells loaded with control solution, and those loaded with CPA. There was no difference between the ratio of the fifth/first KAR-EPSC amplitude (control: 7.5 ± 1.7; *n* = 5; CPA: 6.9 ± 1.2; *n* = 5; *p* > 0.5; data not shown), suggesting no difference in Pr. Thus, the increase in PTP seen in CPA-loaded cells was likely because of depletion of postsynaptic Ca^2+^ stores. Lastly, release of Ca^2+^ from internal stores can be triggered by activation of inositol 1,4,5-trisphosphate receptors (IP_3_Rs; Verkhratsky, 2005), a signaling pathway that has been shown to underlie Ca^2+^ rises in CA3 pyramidal neurons (Kapur et al., 2001) and postsynaptic plasticity (Kwon and Castillo, 2008b) at the MF-CA3 synapse. To examine whether IP_3_Rs played a role in the suppression of MF-PTP, we included heparin (2.5 mg/ml) in the patch pipette. With IP_3_Rs blocked, PTP was increased to a similar level as when cells were loaded with CPA (Fig. 4*D*). Together, these results suggest that IP_3_R-triggered release from internal Ca^2+^ stores contributes to the suppressive effect on PTP.

### Assessing the mechanism underlying MF-PTP suppression

**Figure 5.**
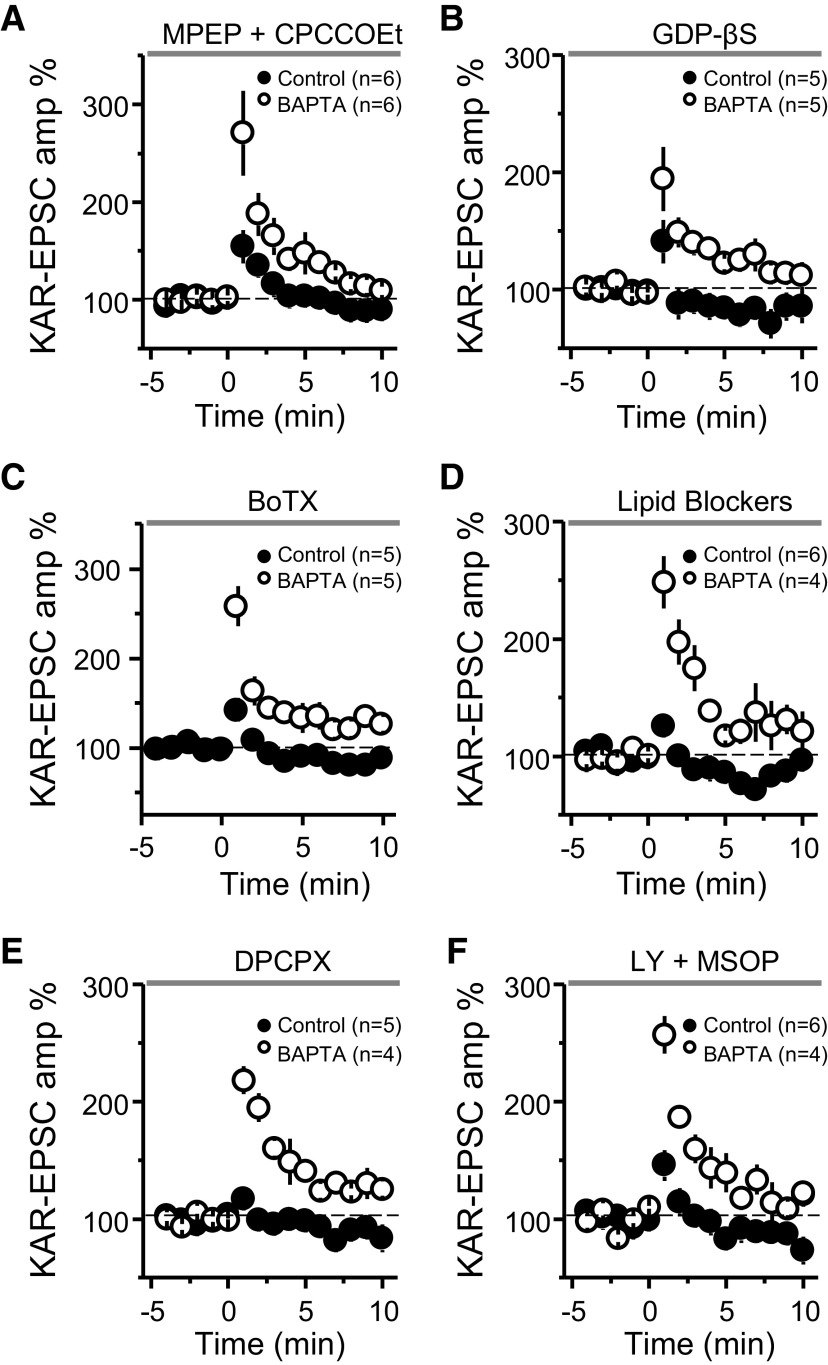
Assessing the mechanism underlying MF-PTP suppression. ***A***, Summary of data of MF-PTP under control (0.1 mm EGTA) and high intracellular buffering conditions (10 mm BAPTA) in the presence of the Group I mGluR antagonists MPEP and CPCCOEt (control: 122 ± 6%; *n* = 6; BAPTA: 208 ± 28%; *n* = 6; control vs BAPTA: *p* < 0.05). ***B***, Summary data of experiments in which control and BAPTA cells were dialyzed with GDP-βS (control-GDP-βS: 106 ± 13%; *n* = 5; BAPTA-GDP-βS: 161 ± 17%; *n* = 5; control-GDP-βS vs BAPTA-GDP-βS: *p* < 0.05). ***C***, Cells dialyzed with BoTX did not exhibit any more PTP than interleaved controls without BoTX (BoTX included in control solution: 116 ± 2%; *n* = 5; BAPTA without BoTX: 189 ± 16%; *n* = 5; control-BoTX vs BAPTA: *p* < 0.01). ***D***, Summary data when a cocktail of lipid blockers (ETYA, indomethacin, and RHC-80267) was included in the intracellular recording solution (control: 105 ± 6% of baseline; *n* = 6; BAPTA: 220 ± 21%; *n* = 4; control vs BAPTA: *p* < 0.001). ***E***, Summary data showing no PTP in control cells after bursting paradigm in the presence of the adenosine 1 receptor antagonist DPCPX, whereas robust PTP observed in BAPTA-dialyzed cells (control: 104 ± 3%; *n* = 5; BAPTA: 191 ± 7%; *n* = 4; control vs BAPTA: *p* < 0.001). ***F***, Summary data showing the effect of bursting paradigm in control and BAPTA-dialyzed cells in the presence of the Group II and III mGluR antagonists LY-341495 and MSOP (control: 121 ± 10%; *n* = 6; BAPTA: 201 ± 10%; *n* = 4; control vs BAPTA: *p* < 0.001). See Extended Data Figure 5-1 for supporting information.

One way that the release of Ca^2+^ from internal stores can be triggered is via activation of Group I mGluRs (i.e., mGluR1 and mGluR5 subtypes). These G-protein-coupled receptors (GPCRs) are likely activated during our PTP induction protocol and have been shown to mobilize Ca^2+^ stores at the MF-CA3 synapse (Kapur et al., 2001). However, with the mGluR5 antagonist MPEP (4 μm) and the mGluR1 antagonist CPCCOEt (100 μm) in the bath solution, a large difference remained between the potentiation observed in control and BAPTA-dialyzed cells (Fig. 5*A*), whereas in separate experiments we found these antagonists greatly reduced the inward current induced by the Group I mGluR agonist DHPG (Extended Data Fig. 5-1*A*). To test for the potential involvement of other GPCRs in mobilizing Ca^2+^ from internal stores (e.g., by activating IP_3_Rs), we used GDP-βS (1 mm), a nonhydrolyzable GDP analog that interferes with G-protein signaling. Including GDP-βS in the recording pipette solution had no apparent effect on the retrograde suppression as we observed significantly less MF-PTP in control compared with BAPTA-dialyzed cells (Fig. 5*B*). As positive control, we found that intracellularly loaded GDP-βS in CA3 pyramidal neurons abolished the outward current induced by the GABA_B_R agonist baclofen (50 μm; Extended Data Fig. 5-1*B*). Together, these results suggest that mobilization of the putative retrograde signal that suppresses MF-PTP requires IP_3_R-mediated release of Ca^2+^ from internal stores, but is independent from the activation of Group I mGluRs or other GPCR-dependent signaling, although we cannot rule out the possibility that G-protein-independent signaling could be involved (Gerber et al., 2007).

Dendrites on postsynaptic neurons have been shown to release retrograde messengers consisting of lipids, gases, peptides, growth factors, and conventional neurotransmitters (Regehr et al., 2009), some of which are released by SNARE-dependent exocytosis. To test whether the putative retrograde signaling suppressing MF-PTP involves vesicular release, we used BoTX, which cleaves synaptobrevin-2, thus eliminating SNARE-dependent exocytosis (Schiavo et al., 2000; Montal, 2010). Adding BoTX (5 nm) to the intracellular solution did not enhance MF-PTP (Fig. 5*C*). In separate, interleaved experiments that served as a positive control, and as previously reported (Lledo et al., 1998), we found that loading BoTx into CA1 pyramidal neurons blocked LTP of AMPAR-mediated transmission (see Materials and Methods; Extended Data Fig. 5-1*C*). These findings argue against SNARE-dependent exocytosis in mediating the putative retrograde signal involved in MF-PTP suppression.

We next examined whether lipids mediate MF-PTP suppression. For instance, endocannabinoids, perhaps the most characterized retrograde signals in the brain (Kano et al., 2009; Castillo et al., 2012), suppress PTP at the parallel fiber-Purkinje cell synapse in the cerebellum by activating presynaptic type 1 cannabinoid receptors (Beierlein et al., 2007). However, these receptors are not expressed at the MF-CA3 synapse in mature animals (Marsicano and Lutz, 1999; Katona et al., 2006; Hofmann et al., 2008; Caiati et al., 2012). To test whether a different lipid signal acting as a retrograde signal, such as arachidonic acid (AA; Carta et al., 2014), or the AA metabolite 12-(S)-HPETE (Feinmark et al., 2003), could suppress MF-PTP, we added a cocktail of inhibitors to the patch pipette solution to inhibit AA and other components of lipid synthesis in the postsynaptic neuron. We included eicosatetraynoic acid (ETYA; 100 μm) and indomethacin (10 μm) to inhibit lipoxygenases and cyclooxygenases (COX 1 and 2), enzymes that catalyze the metabolism of eicosanoids and prostanoids, respectively. We also added RHC-80267 (50 μm) to inhibit diacylglycerol (DAG) lipase. With this combination of lipid inhibitors in the pipette, we continued to see robust MF-PTP in BAPTA-dialyzed cells, but none when the cocktail was included in control cells (Fig. 5*D*). Our results suggest that the putative retrograde signal that suppressed PTP at the MF-CA3 synapse does not depend on these lipid-derived pathways.

Lastly, we explored potential ways by which glutamate release was suppressed during PTP. Presynaptic type 1 adenosine receptors (A1Rs) can tonically inhibit glutamate release at this synapse (Moore et al., 2003; but see Kukley et al., 2005). To test whether these receptors mediate MF-PTP suppression, we used the A1R-selective antagonist DPCPX (200 nm). DPCPX did not alter the robust difference in PTP observed in control versus BAPTA-dialyzed cells (Fig. 5*E*), but significantly increased the amplitude of MF KAR-EPSCs (Extended Data Fig. 5-1*D*), indicating that DPCPX was active and therefore, A1Rs were not underlying the suppression of MF-PTP. Glutamate release from MFs is also blocked by the activation of presynaptic Group II/III mGluRs (Kamiya et al., 1996). While our results using BoTX make it unlikely that these receptors were targeted by glutamate released from the postsynaptic cell, glutamate could have been generated from other sources (e.g., glia). However, in the presence of the Group II and III mGluR antagonists LY 341495 (1 μm) and MSOP (200 μm), respectively, the large difference between control and BAPTA remained (Fig. 5*F*). These antagonists almost completely reverse the DCG-IV-mediated suppression of MF transmission (Extended Data Fig. 5-1*E*). It is therefore unlikely that activation of presynaptic mGluR2/3 underlies the suppression of MF-PTP.

### Strong presynaptic activity is required for retrograde suppression of glutamate release

**Figure 6.**
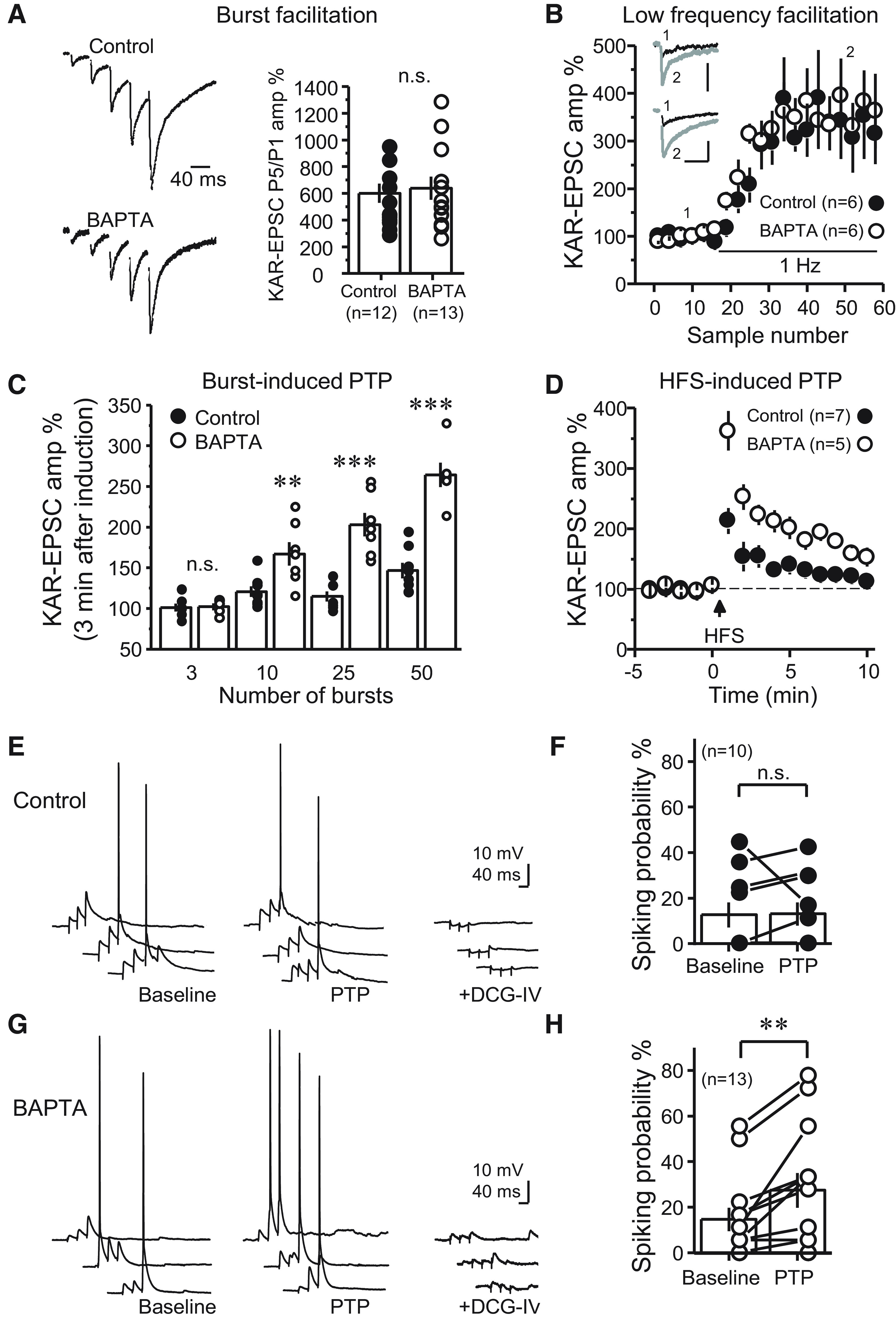
MF-PTP suppression under different patterns of activity. ***A***, Five pulses were delivered to MFs for control and BAPTA-dialyzed cells, and the ratio of the fifth KAR-EPSC to the first was taken (P5/P1 control: 6.0 ± 0.7; *n* = 12; P5/P1 BAPTA: 6.5 ± 0.9; *n* = 13; control vs BAPTA: *p* > 0.5). Representative traces (normalized to the amplitude of first EPSC) are shown on the left side. ***B***, Summary data showing effect of switching basal stimulation frequency from 0.1 to 1.0 Hz in control versus BAPTA-dialyzed cells (LFF control: 341 ± 73% of baseline; *n* = 6; LFF BAPTA: 358 ± 59%; *n* = 6; control vs BAPTA: *p* > 0.5). Insets, Superimposed traces taken at 0.1 and 1.0 Hz. Calibration bars: 40 pA and 40 ms. ***C***, Summary data showing the suppressive effect on MF-PTP induced by different number of bursts. Three bursts, control: 101 ± 5% of baseline; *n* = 7; BAPTA: 102 ± 4%; *n* = 5 (control vs BAPTA: *p* > 0.5). Ten bursts, control: 121 ± 7% of baseline; *n* = 8; BAPTA: 167 ± 14%; *n* = 7 (control vs BAPTA: *p* < 0.01). Twenty-five bursts: control: 115 ± 6% of baseline; *n* = 7; BAPTA: 203 ± 14%; *n* = 7 (control vs BAPTA: *p* < 0.001). Fifty bursts: control: 147 ± 10% of baseline; *n* = 8; BAPTA: 264 ± 15%; *n* = 6 (control vs BAPTA: *p* < 0.001). ***D***, Effect of HFS (100 pulses at 100 Hz, 3×) on MF KAR-EPSCs, with or without BAPTA in the patch pipette (control: 181 ± 20% of baseline; *n* = 7; BAPTA: 282 ± 24%; *n* = 5; control vs BAPTA: *p* < 0.01). ***E–H***, Effects of MF-PTP on CA3 pyramidal neuron firing induced by MF bursting stimulation in current-clamp configuration. No drugs were added to the bath. Control cells loaded with 0.1 mm EGTA (***E***, ***F***) showed no significant increase in spike probability after PTP induction (baseline: 12.6 ± 5.5%; *n* = 10; PTP: 13.07 ± 5%; *n* = 10; baseline vs PTP: *p* > 0.5, Wilcoxon signed-ranked test), whereas in cells loaded with 10 mm BAPTA (***G***, ***H***) PTP enhanced the probability of firing action potentials (baseline: 14.52 ± 5.1%; *n* = 10; PTP: 27 ± 7.5%; *n* = 10; baseline vs PTP: *p* < 0.01, Wilcoxon signed-ranked test). n.s., non-significant; **, *p* < 0.01; ***, *p* < 0.001.

The types of activity under which retrograde suppression of transmitter release manifests could have important implications for the CA3 network. We found that burst-induced facilitation, measured by the ratio of the fifth KAR-EPSC amplitude to that of the first (P5/P1) in a single burst (five pulses, 25 Hz), was not significantly different in control versus BAPTA conditions (Fig. 6*A*). We also examined the effect of postsynaptic Ca^2+^ buffering on low-frequency facilitation (LFF), whereby single KAR-EPSCs were evoked, first during 0.1 Hz basal stimulation, and after switching to 1 Hz. No difference was observed between conditions (Fig. 6*B*). Thus, for these modest increases in activity, postsynaptic Ca^2+^ buffering seemed to have no impact on presynaptic transmitter release. Moreover, because frequency facilitation is highly dependent on the starting Pr (Zucker and Regehr, 2002; Nicoll and Schmitz, 2005; Regehr, 2012), these results argue against a tonic suppression of neurotransmitter release under high postsynaptic Ca^2+^ buffering conditions.

We next delivered multiple bursts to determine how strong the bursting paradigm must be before retrograde suppression of MF-PTP is observed. To this end, we increased the number of bursts while maintaining both the frequency within a burst (50 Hz), as well as between bursts (2 Hz). After three bursts, synaptic responses were similar in both Ca^2+^ buffering conditions (i.e., control and BAPTA), but a difference emerged after 10 bursts (Fig. 6*C*), and a larger difference was also seen after increasing the number of bursts to 50. In BAPTA-dialyzed cells, there was a difference between 3 and 10 (ANOVA, *F* = 21.9; *p* < 0.05; df = 10), 3 and 25 (*p* < 0.001, df = 10), 3 and 50 (*p* < 0.001, df = 9), 10 and 50 (*p* < 0.001, df = 11), and 25 and 50 bursts (*p* < 0.05, df = 11). Together, these data not only uncover the magnitude of MF-PTP in the absence of a retrograde suppressive signal, but also show that in our BAPTA conditions, a longer bursting paradigm induces stronger MF-PTP. The threshold observed with 10 bursts is relatively modest, highlighting that this form of regulation could likely manifest *in vivo*.

We next examined whether stronger activation of MFs could overcome the suppressive retrograde signal. To address this possibility, we delivered HFS consisting of three trains of 100 stimuli (100 Hz within a train; trains separated by 10 s). While a sizeable potentiation was elicited in control cells, the magnitude of MF-PTP was significantly larger in conditions of high Ca^2+^ buffering (Fig. 6*D*). Thus, the putative retrograde signal is strong enough to dampen the PTP evoked even by prolonged high-frequency tetanus.

It has been suggested that the MF-CA3 synapse can operate as a conditional detonator (Treves and Rolls, 1992; Urban et al., 2001; Henze et al., 2002), and a recent study demonstrated that MF-PTP could convert MF-CA3 synapses into full detonators (Vyleta et al., 2016). However, recordings in this study, like many other voltage-clamp studies, were performed under high postsynaptic Ca^2+^ buffering conditions, i.e., 10 mm EGTA in the recording pipette. We therefore reassessed the role of PTP in MF detonation using physiological intracellular Ca^2+^ buffering. To this end, we tested whether PTP induction facilitated the ability of a short MF burst (three stim, 25 Hz) to generate action potentials in the postsynaptic CA3 pyramidal neurons loaded with either 0.1 mm EGTA (control) or 10 mm BAPTA. Consistent with previous findings (Nelson et al., 2003; Roussel et al., 2006), we observed that action potentials were easier to elicit in BAPTA-loaded neurons. A comparable baseline spiking probability between control and BAPTA neurons was achieved by slightly adjusting the stimulus strength and depolarizing the control neurons by ∼10 mV. We found that the spike probability 3 min post-PTP was significantly enhanced in BAPTA but not control cells (Fig. 6*E–H*). These results indicate that the contribution of PTP to MF detonation can be overestimated under high postsynaptic Ca^2+^ buffer recording conditions.

## Discussion

We report here that the Ca^2+^ buffering capacity of the postsynaptic neuron can significantly impact presynaptically expressed PTP at the hippocampal MF-CA3 synapse. Under normal Ca^2+^ buffering capacity, as observed during non-invasive recording conditions (e.g., perforated patch recording) that do not significantly alter the physiological intracellular milieu, we found that a bursting induction protocol designed to mimic *in vivo* activity patterns of GCs triggered a negligible PTP. However, a far greater potentiation was revealed under high postsynaptic Ca^2+^ buffering conditions. Remarkably, increasing the postsynaptic buffer capacity had no significant effect on the basal Pr, arguing against a tonic suppression of neurotransmitter release. The most parsimonious explanation for our findings is the presence of a Ca^2+^-dependent, retrograde signaling mechanism that suppresses PTP. A minimum threshold was required before the phenomenon was observed, above which it operated in a wide range of activity. These results point to a novel, activity-dependent form of negative feedback at the MF-CA3 synapse that may significantly impact DG-CA3 information transfer.

At first glance, our findings contrast starkly with numerous studies that have reported pronounced MF-PTP (for review, see Henze et al., 2000). However, most of these studies elicited MF-PTP with strong repetitive stimulation (e.g., HFS) while monitoring MF transmission with extracellular field recordings. Because of activation of the CA3 network, these experimental conditions not only enable the recruitment of associational-commissural inputs that are commonly interpreted as MF-mediated responses, but also facilitate population spike contamination of (extracellularly recorded) synaptic responses (Henze et al., 2000; Nicoll and Schmitz, 2005). As a result, MF-PTP magnitude can be easily overestimated. Strong MF-PTP was also observed in studies that used more sensitive, single-cell recordings (Zalutsky and Nicoll, 1990; Maccaferri et al., 1998; Vyleta et al., 2016; Vandael et al., 2020), but here again it was typically evoked with a rather non-physiological induction protocol such as HFS. Critically, many such previous studies also loaded postsynaptic neurons with 10 mm EGTA, a standard concentration used in whole-cell studies, both *in vitro* and *in vivo*. Indeed, we also observed striking MF-PTP in those conditions (Fig. 2*B*). Our findings therefore suggest that MF-PTP is tightly controlled when afferent stimulation and postsynaptic Ca^2+^ buffering are set to more physiological levels.

A rise in postsynaptic Ca^2+^ is required for most forms of retrograde signaling (Fitzsimonds and Poo, 1998; Regehr et al., 2009). Consistent with this notion, MF-PTP was suppressed when the postsynaptic cell was loaded with high (millimolar) concentrations of EGTA or BAPTA, or with CPA or heparin, all agents that interfere with intracellular Ca^2+^ rise. To determine the potential source(s) of postsynaptic Ca^2+^ rise involved in the suppression of MF-PTP, we pharmacologically interfered with these sources one by one. We found that IP_3_R-mediated Ca^2+^ release from intracellular stores contributed to the suppression, but that Group I mGluRs and G-protein-coupled signaling alone were insufficient. Importantly, however, we cannot discard synergism of multiple sources leading to the full rise in postsynaptic Ca^2+^ required for retrograde suppression of MF-PTP, including the contribution of VGCCs in a poorly voltage-clamped postsynaptic compartment (Williams and Mitchell, 2008; Beaulieu-Laroche and Harnett, 2018). Interestingly, the endogenous Ca^2+^ buffering power of CA3 pyramidal neurons is the source of some debate, as some have suggested it is higher than that of CA1 cells (Wang et al., 2004), while others report it is similar (Simons et al., 2009). Further complicating this matter, different endogenous buffers and extrusion mechanisms create short-lived Ca^2+^ nanodomains which can limit the interaction between Ca^2+^ and its substrates (Higley and Sabatini, 2008). Our data show little PTP when the intracellular composition was not perturbed in perforated patch mode, and robust PTP when the cell was dialyzed with a high concentration of BAPTA (Fig. 2*C*). This observation would indicate that, regardless of the exact endogenous buffering power of these cells, it is sufficiently low to allow for the postsynaptic neuron to regulate MF-PTP.

Retrograde signaling has been proposed to influence PTP, but typically in the opposite direction to our findings. For example, in the *Aplysia* sensorimotor neuron preparation, injection of BAPTA into the postsynaptic cell depressed PTP (Bao et al., 1997). In the hippocampus, loading CA3 pyramidal neurons with high concentrations of BAPTA (30–50 mm) curtailed PTP and abolished LTP at the MF-CA3 synapse, presumably by blocking the mobilization of a retrograde signal (Yeckel et al., 1999; but see Mellor and Nicoll, 2001). There is also evidence that the receptor tyrosine kinase ephrin B (EphB) and its membrane-bound ligand ephrin B mediate retrograde communication at MF-CA3 synapses, and that interfering with EphB/ephrin B signaling inhibits both PTP and LTP at MF-CA3 synapses (Contractor et al., 2002; Armstrong et al., 2006). Our findings resemble the endocannabinoid-mediated suppression of PTP at the parallel fiber-Purkinje cell synapse via presynaptic type 1 cannabinoid receptors (Beierlein et al., 2007). However, these receptors are not found at MF boutons in the mature brain (Marsicano and Lutz, 1999; Katona et al., 2006; Hofmann et al., 2008; Caiati et al., 2012). While the identity of the putative retrograde signal generated by the postsynaptic neuron remains unidentified, some usual candidates (Regehr et al., 2009) could be discarded. Vesicular, SNARE-dependent exocytosis was ruled out (Fig. 5*C*), indicating that the messenger is likely not a conventional neurotransmitter (e.g., glutamate or GABA). Further evidence against glutamate as a retrograde signaling mediating PTP suppression is the fact that this suppression remains intact after blocking presynaptic Group II and III mGluRs (Fig. 5*F*). Our findings also argue against the role of another putative signal, nitric oxide (NO) given that its synthesis typically requires the activation of NMDARs (Christopherson et al., 1999; Sattler et al., 1999), which were blocked in most of our experiments. We cannot discard the possibility that PTP suppression involves retrograde signaling via synaptic adhesion molecules. It is worth noting that identifying endocannabinoids as the retrograde messengers mediating the well characterized depolarization-induced suppression of inhibition (DSI) took a decade of work by several groups (Barinaga, 2001). Future studies will have to determine the identity of the presumably non-conventional retrograde signal and the presynaptic substrate responsible for curtailing PTP (Regehr, 2012).

Two recent studies suggested that MF-PTP occurs *in vivo* (Vandael et al., 2020) and switches MF-CA3 synapses into full detonators (Vyleta et al., 2016). However, our results indicate that the impact of PTP was likely overestimated because recordings in both studies were performed under high, non-physiological Ca^2+^ buffering conditions (e.g., 10 mm EGTA). PTP has also been reported at MF to inhibitory interneuron synapses (Alle et al., 2001; Mori et al., 2007). Given that MFs make 10 times as many contacts onto inhibitory interneurons as onto CA3 pyramidal cells (Acsády et al., 1998), PTP at MF to interneuron synapses, by presumably activating an inhibitory network, could control the activity of GCs (feed-back inhibition) and detonation of CA3 pyramidal neurons (feed-forward inhibition; Lawrence and McBain, 2003). However, unlike PTP at MF-CA3 synapses, postsynaptic Ca^2+^ buffering had no impact on PTP magnitude at MF to basket cells synapses in the DG, with similarly robust PTP when the interneuron was loaded with 0.1 mm EGTA or 10 mm BAPTA (Alle et al., 2001). Thus, although PTP is observed at distinct MF synapses, the retrograde suppression we report in the present study seems to be unique to the MF-CA3 pyramidal cell synapse.

Relatively strong GC burst activity is needed for engaging suppression of MF-PTP, and the magnitude of this suppression does not seem to be overcome by stronger presynaptic activity (Fig. 6*C*). Thus, PTP suppression may serve as a powerful mechanism of controlling DG-CA3 information transfer following repetitive bursts of GC activity that normally occur during exploratory behaviors (Henze et al., 2002; Pernía-Andrade and Jonas, 2014; Diamantaki et al., 2016; GoodSmith et al., 2017; Senzai and Buzsáki, 2017). Left unchecked, MF-PTP could indirectly lead to runaway activity of the CA3 network and facilitate epileptic activity. Lastly, while the generalizability of PTP suppression remains untested, future studies will be required to determine whether Ca^2+^ indicators widely used *in vitro* and *in vivo* throughout the brain may promote a similar suppression of presynaptic function.

## Synthesis

Reviewing Editor: Katalin Toth, University of Ottawa Decisions are customarily a result of the Reviewing Editor and the peer reviewers coming together and discussing their recommendations until a consensus is reached. When revisions are invited, a fact-based synthesis statement explaining their decision and outlining what is needed to prepare a revision will be listed below. The following reviewer(s) agreed to reveal their identity: NONE.

This study provides novel and unexpected results about the role of calcium buffering in the expression of PTP at hippocampal mossy fiber inputs. Both reviewers found the experimental generally well designed and executed and the data high-quality. However, both reviewers highlight the need for increased clarity on how differences between PTP and LTP (Rev. #2 used the terminology of ‘early’ and ‘late’ PTP) were established. How the authors differentiate between these two needs to be clearly stated and the figures should provide and equally clear picture to the reader. The authors would need to carefully address this critical point to increase the clarity of their manuscript.

The authors are encouraged to visualize individual datapoints on all of their bar graphs and/or use estimation statistics.

Rev. #1.

This manuscript reports that post-tetanic potentiation (PTP) at the mossy fiber-CA3 pyramidal cell synapse is partly controlled by a postsynaptic mechanism. This is a surprising result, as PTP and most forms of short-term plasticity are often thought to have a presynaptic origin. Specifically, the authors uncover that increasing postsynaptic Ca2+ buffering via BAPTA or EGTA dialysis increases PTP magnitude, an observation defying common rules of associative plasticity. The authors find that the stronger PTP observed in the presence of BAPTA leads to enhanced firing likelihood. Finally, the authors experimentally eliminate an array of possible mechanisms, but uncover that PTP critically depends on postsynaptic internal Ca2+ stores. Overall, the data presented appear to be of high quality, including challenging perforated patch experiments. However, I have several criticisms that dampen my enthusiasm for this manuscript.

1) Distinction between PTP and possible long-term plasticity. PTP is generally defined as a short-lasting enhancement of neurotransmitter release which decays back to baseline levels of release. Here, the authors provide no quantification of PTP duration. Unfortunately, in several examples presented, the PTP in presence of BAPTA doesn’t return to baseline over the 10 minutes time course presented (Figs. 1a, 2a, 2c, 3a, 3b, 3c, 4a, 6d), and sometimes shows very little signs of returning to baseline, obviously reminiscent of long lasting plasticity. Therefore, I am intrigued by whether long-term plasticity mechanisms are involved in the presence of BAPTA, a possibility given the author’s results and the following two papers. First, an enhancement of LTP by postsynaptic BAPTA inclusion might make sense at the MF-CA3 synapse, given that hyperpolarization paired with presynaptic activity potentiates MF inputs (Sokolov et al., 2003). Second, relatively mild stimulation protocols evoke LTP when the CA3 cell membrane potential is left to fluctuate during MF stimulation at this synapse (Jaffe and Johnston, 1990). In the present manuscript, it seems that PTP was induced in the current-clamp configuration in at least some experiments. From the Results section:

-The PTP induction protocol was delivered in current-clamp configuration so that CA3 cells were able to fire freely.

But there is a possible contradiction about how PTP was induced, which would benefit from additional clarification. From the Methods section:

-The burst protocol was delivered while cells were voltage-clamped at -60 mV, except for one experiment in which the induction was performed in current-clamp (Fig. 1a,c and Fig. 6e-h).

Overall, given the results presented here, it is unclear whether the authors are examining PTP or long-term plasticity. In addition, involvement of long-term plasticity mechanisms is plausible given the experimental manipulations performed and previous literature at the MF-CA3 synapses.

2) The data in Fig. 6e-h is unreliable and cannot be interpreted. The authors make the case that in absence of BAPTA, PTP does not increase firing likelihood. However, the comparison is flawed because of a floor effect in control, where the likelihood of baseline firing is set at 0% before PTP. Therefore, it is likely that the full effect of PTP on firing is obscured under this condition. In contrast, the experiments performed in BAPTA were adjusted to have firing probability of ˜25% at baseline before PTP. In defense of this, the authors note that (page 16, line 14): ‘Eliciting action potentials in BAPTA neurons was easier than in control neurons, which could be due to changes in excitability (Nelson et al., 2003; Roussel et al., 2006).’ This argument renders the control vs. BAPTA comparison difficult, but the floor effect described above makes it invalid. it is also apparent that: 1) the first EPSP is of larger amplitude in presence of BAPTA (panel g) vs. control (panel e); and 2) EPSP appear to summate differently.

3) All data are presented as normalized to a control period, except for data presented in the Extended Figures. While this allows for comparison between different cells and different experimental groups, it is necessary to present raw EPSC measurements as well. This would help the reader to understand if the variety of treatment performed have any effect on basal synaptic transmission.

Rev.#2.

This work report an interesting and unexpected finding, ie the Ca2+ buffering capacity of postsynaptic CA3 hippocampal neurons impacts presynaptically-expressed mossy fiber post-tetanic potentiation (PTP). Because of this, the author(s) argue for the involvement of a retrograde transmitter. They perform several pharmacological experiments and are able to exclude some candidates. In general, experiments are carefully designed, nicely executed, and appropriately discussed. The quality of the recordings is very high, which builds confidence in the results. In conclusion, I think this is an interesting study that should attract the attention of many synaptic physiologists, given its implications for a yet unidentified retrograde neurotransmitter at the MF-CA3 synapse.

There are just a few points that require the attention of the author(s).

1) From the initial figures, the impression is that PTP lasts for the entire duration of the plots (10 min, see the difference between control and BAPTA recordings in Figs 1a, 1c, and 2a, for example). Therefore, one would expect that manipulations affecting PTP should impact both the "early" (the authors use the first 3 min following the induction protocol to measure PTP) and the "late" phase of the phenomenon. Some figures, however, are a little difficult to interpret. For example, in Figures 4b and 4c, CPA or heparin inclusion in the pipette reveal only the "early" phase of PTP, whereas the "late" phase seems to be missing. Could the author(s) please discuss this? Also, I notice that in Figure 4b, the PTP protocol in experiments using control pipettes seems to trigger a strong depression of synaptic transmission. Could the author(s) please mention/discuss this additional surprising finding?

2) Methods. "Data that did not display a normal distribution". How was this evaluated? Could the author(s) please clarify?

3) Methods. In the experiments using picrotoxin, how was epileptiform activity avoided?

4) Figure 6a. The scale bar is missing.

5) Page 10, line 26. "burs" should be burst

## Author Response

Makani et al, "Retrograde suppression of post-tetanic potentiation at the mossy fiber to CA3 pyramidal cell synapse"

We thank the reviewers for their constructive comments and helpful suggestions, and the Reviewing Editor for handling our manuscript. We are also glad to know the reviewers understand our study provides novel and unexpected results and they found that our experiments are well designed and generated high-quality data. Below, please find our point-by-point response to the reviewers' comments. Changes in the manuscript are marked in blue.

Reviewer #1:

We thank Reviewer #1 for his/her critical reading of our manuscript. This reviewer indicates our findings are surprising and that the data presented "appear to be of high quality". He/she also raises several criticisms that we attempted to address in this revised manuscript.

1) Distinction between PTP and possible long-term plasticity. PTP is generally defined as a short-lasting enhancement of neurotransmitter release which decays back to baseline levels of release. Here, the authors provide no quantification of PTP duration. Unfortunately, in several examples presented, the PTP in presence of BAPTA doesn’t return to baseline over the 10 minutes time course presented (Figs. 1a, 2a, 2c, 3a, 3b,

3c, 4a, 6d), and sometimes shows very little signs of returning to baseline, obviously reminiscent of long lasting plasticity. Therefore, I am intrigued by whether long-term

plasticity mechanisms are involved in the presence of BAPTA, a possibility given the

author’s results and the following two papers. First, an enhancement of LTP by postsynaptic BAPTA inclusion might make sense at the MF-CA3 synapse, given that

hyperpolarization paired with presynaptic activity potentiates MF inputs (Sokolov et al.,

2003). Second, relatively mild stimulation protocols evoke LTP when the CA3 cell membrane potential is left to fluctuate during MF stimulation at this synapse (Jaffe and Johnston, 1990). In the present manuscript, it seems that PTP was induced in the current-clamp configuration in at least some experiments.

...

Overall, given the results presented here, it is unclear whether the authors are examining PTP or long-term plasticity. In addition, involvement of long-term plasticity mechanisms is plausible given the experimental manipulations performed and previous literature at the MF-CA3 synapses.

PTP at the MF-CA3 synapse is not only particularly robust but also longer lasting. While at most synapses PTP barely last a few minutes (∼3 min), MF-PTP may last 15 min or more (Langdon et al., 1995; Henze et al., 2000). As indicated by the reviewer, in some of our experiments PTP does not seem return to baseline within 10 min. However, this is not the case for several other experiments (see for example Figs. 1c, 2b, 4c, 4d, 5a and

5b). Given the transient nature of PTP, we focused our attention on the first three minutes post induction. It is likely that our bursting protocol induced PTP of variable duration. Note that in Fig. 6d we used a stronger induction protocol (HFS), which is expected to induce a stronger and longer lasting PTP. To directly address the reviewer’s comment, we examine whether BAPTA-loaded cells could facilitate the induction of MF-

LTP by extending our recordings (e.g. 20 minutes post-induction). As shown in Extended Figure 2d, we found that our bursting induction protocol did not induce any LTP in BAPTA-loaded neurons.

From the Results section:

-The PTP induction protocol was delivered in current-clamp configuration so that CA3 cells were able to fire freely.

But there is a possible contradiction about how PTP was induced, which would benefit from additional clarification. From the Methods section:

-The burst protocol was delivered while cells were voltage-clamped at -60 mV, except for one experiment in which the induction was performed in current-clamp (Fig. 1a,c and

Fig. 6e-h).

We thank the reviewer for pointing out our error. The revised text (Methods, page 5, lines 24-25) was modified as follows: "The bursting induction protocol was delivered while cells were voltage-clamped at -60 mV, except for the experiments included in Figures 1a,c, 2c and Fig. 6e-h in which PTP was induced in current-clamp."

2) The data in Fig. 6e-h is unreliable and cannot be interpreted. The authors make the case that in absence of BAPTA, PTP does not increase firing likelihood. However, the comparison is flawed because of a floor effect in control, where the likelihood of baseline firing is set at 0% before PTP. Therefore, it is likely that the full effect of PTP on firing is obscured under this condition. In contrast, the experiments performed in BAPTA were adjusted to have firing probability of ˜25% at baseline before PTP. In defense of this, the authors note that (page 16, line 14): ‘Eliciting action potentials in BAPTA neurons was easier than in control neurons, which could be due to changes in excitability (Nelson et al., 2003; Roussel et al., 2006).’ This argument renders the control vs. BAPTA comparison difficult, but the floor effect described above makes it invalid. it is also apparent that: 1) the first EPSP is of larger amplitude in presence of BAPTA (panel g) vs. control (panel e); and 2) EPSP appear to summate differently.

The reviewer raises an important point. In response, we have repeated our experiments under conditions in which both the EPSP amplitude and baseline firing probability were similar in Control and BAPTA neurons. We achieved this goal by adjusting the stimulus strength and by slightly depolarizing control neurons (∼10 mV). This information is included in Methods (page 16, lines 19-21). Our new results confirm that although the baseline spiking probability was almost identical (∼20%) in control and BAPTA conditions, the PTP induction protocol did not increase firing in control neurons.

The reviewer also noted that the EPSP amplitude and summation could be different in Control vs BAPTA neurons. It is important to note that the experimenter sets up the magnitude of the baseline responses such that these responses between Control and BAPTA neurons are comparable before delivering the PTP induction protocol. We have verified the EPSP amplitude (first EPSP) from all neurons included in Fig. 6e-h and found no significant difference between control and BAPTA-loaded neurons (Control: 6.7

{plus minus} 1.6 mV; n = 10; BAPTA: 7.16 {plus minus} 1.35 mV; n = 11; p > 0.5, two-sample t-test). We also found a slightly faster EPSP decay in BAPTA-loaded neurons compare to control (control: 73.8 {plus minus} 8.0 ms; n = 7; BAPTA: 48.5 {plus minus} 5.3 ms; n = 11; p = 0.015, two-sample t- test). Despite a potentially stronger temporal summation in control than in BAPTA- loaded neurons, PTP induction did not increase firing probability in control neurons.

3) All data are presented as normalized to a control period, except for data presented in the Extended Figures. While this allows for comparison between different cells and different experimental groups, it is necessary to present raw EPSC measurements as well. This would help the reader to understand if the variety of treatment performed have any effect on basal synaptic transmission.

We agree in that information about raw EPSC amplitudes used in our study should be provided to the reader. Again, we would like to highlight that, in an attempt to elicit similar responses across conditions, it is the experimenter who sets up the magnitude of the baseline responses. Even so, some unexpected bias could have affected our results. To discard this possibility, we analyzed the amplitude of baseline synaptic responses (both AMPAR and KAR-mediated EPSCs) across experiments. We found no significant difference between control and BAPTA neurons (see Extended Figure 2c), strongly suggesting that changes in the magnitude of the pre-PTP synaptic responses may not account for our main observations.

Reviewer #2:

This reviewer thinks we report "an interesting and unexpected finding", and that "the quality of the recordings is very high, which builds confidence in the results." We thank the reviewer for these positive remarks. He/she finds "just a few points" that require our attention.

1) From the initial figures, the impression is that PTP lasts for the entire duration of the plots (10 min, see the difference between control and BAPTA recordings in Figs 1a, 1c, and 2a, for example). Therefore, one would expect that manipulations affecting PTP should impact both the "early" (the authors use the first 3 min following the induction protocol to measure PTP) and the "late" phase of the phenomenon. Some figures, however, are a little difficult to interpret. For example, in Figures 4b and 4c, CPA or heparin inclusion in the pipette reveal only the "early" phase of PTP, whereas the "late" phase seems to be missing. Could the author(s) please discuss this? Also, I notice that in Figure 4b, the PTP protocol in experiments using control pipettes seems to trigger a strong depression of synaptic transmission. Could the author(s) please mention/discuss this additional surprising finding?

We thank the reviewer for his/her insightful comments. We acknowledge that in some of our experiments PTP does not return to baseline within 10 min. However, this is not the case for several other experiments (see for example Fig 1c, 2b, 4c, 4d, 5a and 5b). As the reviewer rightly indicates, the time course of this form of short-term plasticity is variable. To better assess the time course of PTP, we extended our recordings for 20 minutes post-induction (see new Extended Figure 2d). From these recordings it is hard to identify and early and late phase. On the other hand, CPA and heparin seem to rescue an early phase only, suggesting -but not demonstrating- the existence of two mechanistically distinct PTP phases. As for the depression reported in Fig. 4b, there is evidence that Ni2+ reduces both PTP and LTP at MF-CA3 synapses by suppressing presynaptic Ca2+ influx through R-type channels (Breustedt et al., 2003; Dietrich et al.,

2003). To demonstrate the effects of Ni2+ on PTP, we replaced the nifedipine experiment with a new experiment using the L-type VGCC blocker nimodipine (Fig. 4a) -i.e. same

inhibitor used in combination with Ni2+. Intriguingly, MF-PTP in the presence of Ni2+ and nimodipine was followed by synaptic depression. Presumably by reducing presynaptic Ca2+ influx, Ni2+ could have enabled the induction of some form of synaptic depression, a possibility that we will address in a separate study. It is worth noting that despite the robust presynaptic action of Ni2+, intracellular BAPTA was still able to enhance MF-PTP, making it unlikely that postsynaptic R-type and T-type channels may contribute significantly to MF-PTP suppression.

2) Methods. "Data that did not display a normal distribution". How was this evaluated? Could the author(s) please clarify?

Normal distribution was assessed using the Shapiro-Wilk test. We have clarified this point in Methods (page 7, lines 6-8): "Data that did not display a normal distribution using the Shapiro-Wilk test were compared using the non-parametric test Mann-Whitney and Wilcoxon Signed Ranked test for unpaired and paired conditions, respectively."

3) Methods. In the experiments using picrotoxin, how was epileptiform activity avoided?

The presence of picrotoxin was accompanied with a saturating concentration of the selective non-competitive AMPAR antagonists GYKI53655 (30 μM) or LY303070 (15

μM), except for experiments included in Fig. 1a,b where AMPAR-mediated responses were recorded in a low concentration of GYKI53655 (1 μM), which is sufficient to reduce

CA3 network activity (Kwon and Castillo, 2008).

4) Figure 6a. The scale bar is missing.

Traces in Fig 6a are normalized and for this reason we did not show an amplitude scale bar. The Figure Legend now reads as follows: "Representative traces (normalized to the amplitude of first EPSC) are shown on the left side."

5) Page 10, line 26. "burs" should be burst

Done. Thank you.

References

Breustedt J, Vogt KE, Miller RJ, Nicoll RA, Schmitz D (2003) Alpha1E-containing Ca2+ channels are involved in synaptic plasticity. Proc Natl Acad Sci U S A 100:12450-12455.

Dietrich D, Kirschstein T, Kukley M, Pereverzev A, von der Brelie C, Schneider T, Beck H (2003) Functional specialization of presynaptic Cav2.3 Ca2+ channels. Neuron 39:483-496.

Henze DA, Urban NN, Barrionuevo G (2000) The multifarious hippocampal mossy fiber pathway:

a review. Neuroscience 98:407-427.

Kwon HB, Castillo PE (2008) Role of glutamate autoreceptors at hippocampal mossy fiber synapses. Neuron 60:1082-1094.

Langdon RB, Johnson JW, Barrionuevo G (1995) Posttetanic potentiation and presynaptically induced long-term potentiation at the mossy fiber synapse in rat hippocampus. J Neurobiol 26:370-385.
